# Neuraminidase-Dependent Degradation of Polysialic Acid Is Required for the Lamination of Newly Generated Neurons

**DOI:** 10.1371/journal.pone.0146398

**Published:** 2016-01-05

**Authors:** Mari Sajo, Hiroki Sugiyama, Hideaki Yamamoto, Takashi Tanii, Norio Matsuki, Yuji Ikegaya, Ryuta Koyama

**Affiliations:** 1 Laboratory of Chemical Pharmacology, Graduate School of Pharmaceutical Sciences, The University of Tokyo, Tokyo, Japan; 2 Frontier Research Institute for Interdisciplinary Sciences, Tohoku University, Miyagi, Japan; 3 Faculty of Science and Engineering, Waseda University, Tokyo, Japan; Universidade de São Paulo, BRAZIL

## Abstract

Hippocampal granule cells (GCs) are generated throughout the lifetime and are properly incorporated into the innermost region of the granule cell layer (GCL). Hypotheses for the well-regulated lamination of newly generated GCs suggest that polysialic acid (PSA) is present on the GC surface to modulate GC-to-GC interactions, regulating the process of GC migration; however, direct evidence of this involvement is lacking. We show that PSA facilitates the migration of newly generated GCs and that the activity of N-acetyl-α-neuraminidase 1 (NEU1, sialidase 1) cleaves PSA from immature GCs, terminating their migration in the innermost GCL. Developing a migration assay of immature GCs *in vitro*, we found that the pharmacological depletion of PSA prevents the migration of GCs, whereas the inhibition of PSA degradation with a neuraminidase inhibitor accelerates this migration. We found that NEU1 is highly expressed in immature GCs. The knockdown of NEU1 in newly generated GCs *in vivo* increased PSA presence on these cells, and attenuated the proper termination of GC migration in the innermost GCL. In conclusion, this study identifies a novel mechanism that underlies the proper lamination of newly generated GCs through the modulation of PSA presence by neuronal NEU1.

## Introduction

The lamination of neuronal cell bodies and fibers is a characteristic feature of cortical organization. It has been proposed that disordered neuronal migration and differentiation result in a disturbed lamination of neurons that increases the risk of neurological disorders, including epilepsy [1]. In the dentate gyrus, following the initial formation of the infra- and suprapyramidal blade of granule cell layer (GCL) [2], newly generated granule cells (GCs) are functionally incorporated into the innermost region of a densely packed cell layer throughout lifetime [3,4]. It has been suggested that the laminated organization of the GCL is necessary to receive highly laminated afferent inputs in the dentate gyrus [5] and that the abnormal positioning of GCs is associated with temporal lobe epilepsy [6]. The overwhelming majority of GCs are created during the first two weeks after birth [7]. Therefore, it is important to clarify the mechanisms underlying the migration and incorporation of newly generated GCs to the appropriate cell layer during the postnatal period.

In the present study, we investigated the role of polysialic acid (PSA) in the migration and positioning of GCs in the postnatal dentate gyrus. PSA is a long linear homopolymer of alpha-2, 8-linked sialic acid residues that is added to the fifth Ig-like domain of the neural cell adhesion molecule (NCAM) by two Golgi-associated polysialyltransferases, ST8SiaIV and ST8SiaII [8,9]. The presence of PSA on the cell surface reduces cell-to-cell adhesion because of its large volume [10], and its capacity to attenuate *trans* binding between adhesive molecules [11] has been suggested to facilitate the migration of PSA-positive neuronal precursors. In the rostral migratory stream, the migration of PSA^+^ neuroblasts is attenuated in PSA-deficient mice or when PSA is enzymatically removed using Endoneuraminidase-N (Endo-N) [12–14]. However, the fundamental mechanism behind the regulation of PSA presence is unknown. The specific localization of PSA in the immature but not mature GCs leads us to hypothesize that PSA plays a role in the migration and positioning of newly generated GCs. Using *in vitro* and *in vivo* analyses, we identified that an unexpected function of the sialidase NEU1 in immature GCs is the cell-autonomous regulation of PSA presence during GC migration and positioning.

## Materials and Methods

### Ethical statement

Animal experiments were performed with the approval of the animal experiment ethics committee at the University of Tokyo (approval number: 24–6) and according to the University of Tokyo’s guidelines for the care and use of laboratory animals. Here we confirm that the animal experiment ethics committee at the University of Tokyo specifically approved this study under the approval number 24–6.

### Pharmacological agents

NeuAc2en (250 μM; Nacalai Tesque, Kyoto, Japan) and Endoneuraminidase-N (Endo-N; 0.007 U/ml; AbCys, Paris, France) were used in this study.

### Animals

Sprague-Dawley rat pups with their mother (SLC, Shizuoka, Japan) were housed in cages in standard laboratory conditions (a 12-h light/dark cycle, free access to food and water). All efforts were made to minimize the animals' suffering and the number of animals used. For preparing hippocampal slice cultures, the rat pups were decapitated after deeply anesthetized on ice, and their mothers were euthanized by isoflurane.

### Organotypic culture of hippocampal slices

Entorhino-hippocampal slice cultures (300 μm thick) were prepared from postnatal day 14 (P14) Sprague-Dawley rats (SLC, Shizuoka, Japan) as described previously [15]. Briefly, rat pups were deeply anesthetized with isoflurane and decapitated, and their brains were removed and horizontally cut into 300-μm-thick slices using a DTK-1500 vibratome (Dosaka, Kyoto) in aerated, ice-cold Gay’s balanced salt solution. Entorhino-hippocampal slices were placed on an Omnipore^®^ membrane filter (JHWP02500, Φ25 mm; Millipore) that was laid on an O-shaped plastic plate. Doughnut plates were inserted into six-well plates that were each filled with 1 ml of culture medium, out of which 0.9 ml of medium was changed every 3.5 days. The medium consisted of 50% minimal essential medium, 25% Hank’s balanced salt solution, and 25% horse serum (Thermo Fisher Scientific, Waltham, MA, USA), supplemented with 33 mM glucose. Finally, the slices were cultured at 37°C in a humidified incubator with 5% CO_2_ and 95% air. To label proliferating cells, the S-phase marker 5-bromo-2’-deoxyuridine (BrdU; 100 mg/kg; Sigma, St. Louis, MO) was subcutaneously injected into rat pups 24 h before slice preparation (P13).

### Explant culture of the dentate hilus

First, 300-μm thick hippocampal slices were prepared from P4-6 Sprague-Dawley rats as described above. Neonatal-generated GCs extensively proliferate and migrate inthe dentate hilar region during the postnatal period. Therefore, the dentate hilus was carefully dissected using a microscalpel (Microfeather P-730; Feather safety razor, Osaka, Japan) under microscopic control to exclude the contamination of mature GCs and pyramidal cells. Each dentate hilar explant was placed on a 13-mm glass coverslip that was pre-coated with poly-l-lysine (Sigma) at 4°C overnight and subsequently coated with laminin (20 μg/ml; Sigma) for 2 h in an incubator with a humidified atmosphere of 5% CO_2_ and 95% air. To adhere to the coated glass, the explants were exposed to 5 μl Neurobasal medium (Life Technologies, Gaithersburg, MD) that was supplemented with 0.5 mM l-glutamine and 2% B-27 (Life Technologies) for 30 min in the incubator. Finally, the coverslips containing the explants were immersed in 400 μl of Neurobasal/B27 medium and cultured for 24 h in the incubator.

### Time-lapse imaging of GC migration

For time-lapse imaging of the GC migration, the hilar explants were placed in the hole of a poly (dimethylsiloxane) (PDMS) well (see the following methods) for stabilization and cultured on a poly-l-lysine- and laminin-coated glass in a 35 mm glass bottom dish. CellVoyager (TM) CV1000 confocal system with a 20× objective (Yokogawa, Tokyo, Japan), which was equipped with an incubation system, was used for time-lapse imaging. Images were taken every 30 minutes for up to 10 hours at 12 h after the preparation of explant culture.

### PDMS well

For time-lapse imaging, hydrophobic PDMS wells were used to stabilize the hilar explants on glass cover slips. Briefly, a curing agent and PDMS prepolymer (SYLGARD 184 Silicone Elastomer Kit, Dow Corning, Midland, MI) were thoroughly mixed in a 1: 20 weight ratio to prepare a PDMS block. Then PDMS slices (width: 1 cm; length: 1 cm; thickness: 200 μm) were cut out using a vibratome (Dosaka). In the center of PDMS slices, a hole (diameter: 6 mm), in which the explants were placed, was punched out and a channel (width: 2–3 mm) that connects the hole to the outside culture medium was made. These PDMS wells were sterilized with 70% ethanol before use.

### Transfection of primary GC cultures

Primary cultures of dissociated GCs from P6 Sprague-Dawley rats were generated as described previously [16]. At 2 DIV, cultured GCs were transfected by lipofection using Lipofectamine 2000 (Life Technologies). The ratio of Neu1 shRNA plasmids to Lipofectamine 2000 was 1 μg per 3 μl. After 1 h, the medium was completely removed and replaced.

### Construction of retroviral vector for Neuraminidase 1 shRNA

To knock down Neuraminidase1, short hairpin RNA (shRNA) sequences that targeted rat Neuraminidase1 were incorporated into the retroviral vector pSIREN-RetroQ-ZsGreen (TaKaRa Bio, Shiga, Japan). In this vector, ZsGreen, a green fluorescent protein, is expressed under the control of the CMV promoter. Neuraminidase1 shRNA (Neu1 shRNA) was co-expressed under the control of the human U6 promoter. The target sequence (5’-GGTGAACGACGTAGACACA-3’; TA0732-1-B) was selected by the Dragon Genomics Center (TaKaRa Bio). As a negative control, we used a non-targeting shRNA (5’-TCTTAATCGCGTATAAGGC-3’) that was generated by the manufacturer (TaKaRa Bio).

### Retrovirus preparation

Retroviruses were produced as described previously [17]. The final titer was approximately 10^8^ PFU/ml, as determined in NIH/3T3 cells. To infect newly generated GCs *in vivo*, P0 rats that were deeply anesthetized on ice were stereotactically injected with 0.5 μl of retrovirus into the dentate gyrus region (anteroposterior = 1.1 mm from lambda; lateral = ± 1.6 mm; ventral = 2.0 mm). After the retrovirus infection, physical condition of pups were monitored daily until they were perfused at P14 for immunohistochemistry. In this study, none of the rat pups used became severely ill or died at anytime prior to the experimental endpoint (P14) because of the retrovirus infection. At P14, the rat pups infected with retrovirus were deeply anesthetized with isoflurane and decapitated for immunohistochemistry.

To infect newly generated GCs in slice cultures, 0.5 μl of retrovirus solution was dropped onto the dentate gyrus region at 0 DIV.

### Immunohistochemistry

Experimental animals were anesthetized with diethyl ether (Wako, Osaka, Japan) and perfused transcardially with cold PBS followed by 4% paraformaldehyde (PFA). Hippocampal sections (200 μm-thick) were prepared using a Zero-1 vibratome (Dosaka, Kyoto, Japan). *In vitro* samples were fixed in 4% PFA at 4°C for 24 h. Immunohistochemistry experiments were performed as described previously [17].

The primary antibodies and concentrations included rabbit anti-Prox1 (1:5,000; Millipore, Billerica, MA), mouse IgM anti-PSA (12E3; 1:2,000; a generous gift from Dr. Seki) [18,19], rat anti-BrdU (1:1,000; AbD Serotec, Oxford, UK), mouse anti-Prox1 (1:2,000; Millipore), mouse anti-Glial fibrillary acidic protein (GFAP; 1:1,000; Millipore), mouse anti-beta III Tubulin (TuJ1; 1:500; abcam), and rabbit anti-Neuraminidase1 (NEU1; 1:1,000; Rockland, Philadelphia, PA). The secondary antibodies, goat anti-rabbit IgG Alexa 488 and 594, goat anti-mouse IgG Alexa 594, and goat anti-mouse IgM Alexa 488, were purchased from Life Technologies and used at a concentration of 1:500. To visualize F-actin, some samples were traced with rhodamine-conjugated phalloidin (1:100; Life Technologies). Fluorescence signals were detected using an MRC-1024 confocal imaging system (Bio-Rad, CA, USA) with 20× (N.A., 0.75) and 60× (N.A., 1.20) objectives (Nikon, Tokyo, Japan) and a SP5 confocal microscope with a 40× (N.A., 1.25) objective (Leica, Wetzlar, Germany). PSA and NEU1 levels in the GCs were measured by analyzing the immunofluorescence intensity in Prox1^+^ cells. For each sample, the background fluorescence was obtained from 5 adjacent 20 × 20-μm^2^ areas outside the slice. The values for the background intensity were averaged, and the intensity was expressed as follows: (intensity in GC—background intensity)/(background intensity).

### Data Representation and Statistical Analysis

The data are presented as the mean ± standard error of the mean (SEM) from at least three independent experiments. A student’s *t* test or Tukey’s test was performed for statistical analyses unless otherwise described in the figure legends. In all experiments, the samples and cells were randomly selected and analyzed in a double-blind manner.

## Results

**Fig 1:**
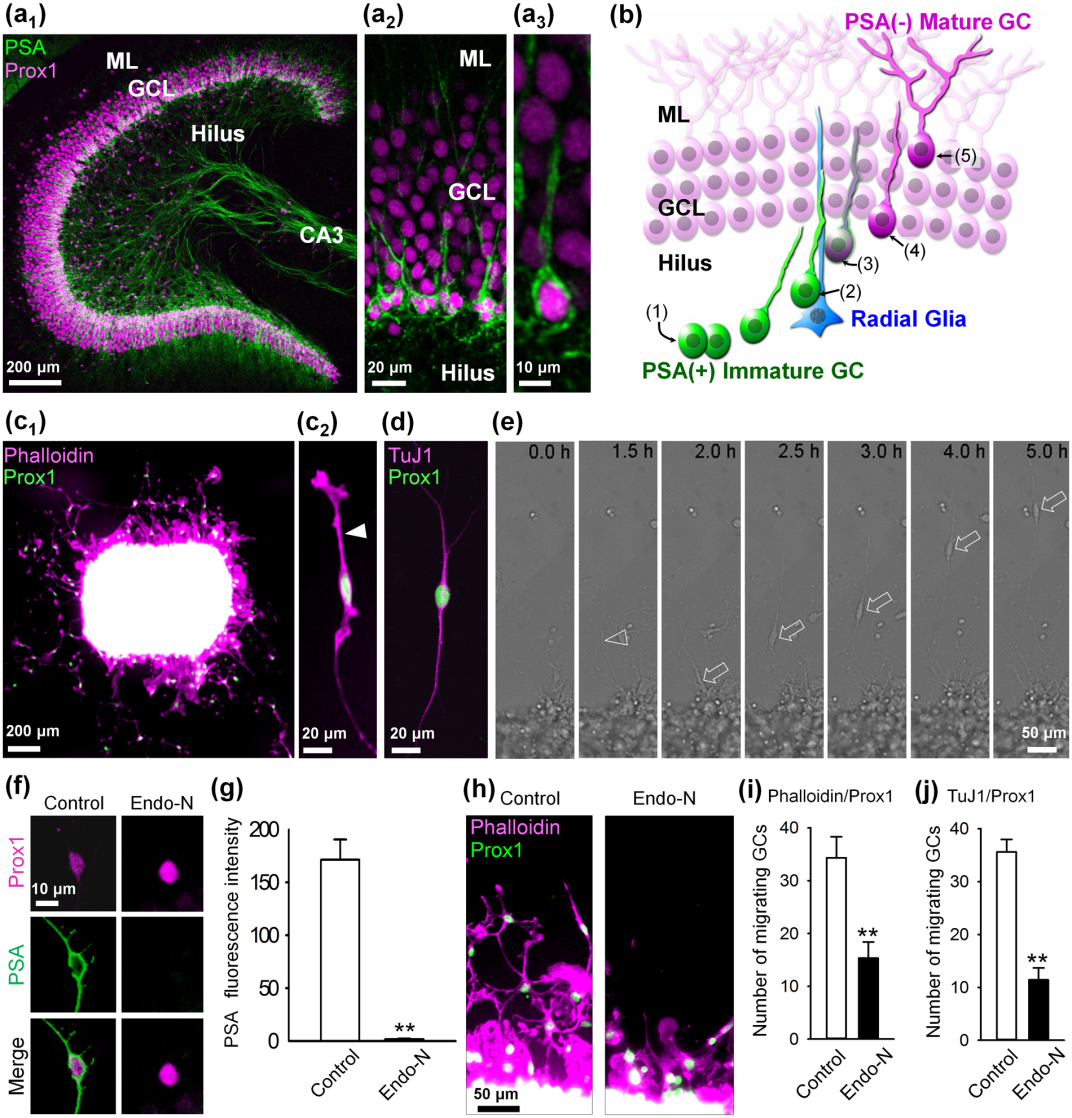
PSA is required for GC migration. (**a**) Representative confocal images of a whole dentate gyrus (**a**_**1**_), the GCL (**a**_**2**_), and a PSA^+^ GC (**a**_**3**_) from a P14 rat hippocampal slice that is immunostained for PSA and Prox1 are shown. The PSA^+^ immature processes of immature GCs that extend into the GCL were observed (**a**_**2**_, **a**_**3**_). ML: molecular layer. (**b**) A schematic diagram showing the maturing process of GCs, which consists of proliferation (1), migration along radial glia (2), degradation of PSA (3), lamination in the GCL (4), and final maturation, including dendritic extension (5). Our hypothesis is that PSA (shown in green) is cleaved from immature GCs during their lamination in the GCL. (**c**) The explant cultures of the dentate hilus from P6 rats were immunostained for Prox1 at 1 DIV. Actin was labeled with rhodamine-phalloidin (**c**_**1**_). A typical migrating GC that possessed a leading process (arrowhead) with a growth cone in the explant culture system is shown (**c**_**2**_). (**d**) Representative image of migrating GCs immunostained for TuJ1 and Prox1. (**e**) Representative time-lapse images of a migrating GC that emanated from the edge of the explant. Images were taken every 30 min at 12 hours after the preparation of explant cultures. An arrowhead at 1.5h indicates a leading process of the observed migrating GC (arrow). Images (**f**) and quantification (**g**) of PSA presence on migrating GCs cultured with or without Endo-N at 1 DIV was determined. **P < 0.01 vs. control, n = 21–22 GCs. Images (**h**) and the number (**i**) of Phalloidin^+^ and Prox1^+^ GCs migrating out from the explants cultured with or without Endo-N at 1 DIV were also obtained. **P < 0.01 vs. control, n = 6–9 explants. (**j**) The number of TuJ1^+^ and Prox1^+^ GCs migrating out from the explants cultured with or without Endo-N at 1 DIV. **P < 0.01 vs. control, n = 5 explants.

Immunolabeling of P14 rat hippocampal slices for PSA, which is a reliable immature GC marker [19], and the developmentally broad GC marker Prox1 revealed the presence of PSA on GCs that are located in the innermost region of the GCL where newly generated GCs proliferate [3]; however, PSA presence was not observed on GCs in the middle and outer GCL (Fig 1a). In addition, these PSA^+^ GCs possessed immature processes with few branches (Fig 1a_2_ and 1a_3_), which is a typical feature of newly generated GCs. Although the role of PSA in GC lamination during development has been unclear, we hypothesized that the superficial localization of PSA on newly generated GCs facilitates their migration, and the degradation of PSA allows these cells to attach to PSA^-^ mature GCs via adhesive molecules (Fig 1b).

To test this hypothesis, we first developed an explant culture system of the hilus, a region where GCs are generated and proliferate during early postnatal periods [2,7] (Fig 1c–1e). We then examined the effect of PSA depletion on GC migration (Fig 1f–1i). Immunocytochemical and time-lapse analyses revealed that a number of Prox1^+^ GCs migrated out from the explant at 1 DIV (Fig 1c–1e). We further confirmed that migrating Prox1^+^ GCs were also immunopositive for TuJ1 (class III beta-tubulin specific for neurons), a conventional migrating neuronal marker (Fig 1d). These cells possess the characteristics of migrating neuronal precursors, including leading processes (Fig 1c_2_, arrow) and strong PSA presence (Fig 1f, control). The application of Endo-N, a phage enzyme that specifically cleaves alpha-2, 8-linked PSA [20], significantly reduced PSA presence in GCs (Fig 1f and 1g). Furthermore, Endo-N inhibited the migration of GCs and retained the cells within the phalloidin-positive edge of the explant (Fig 1h). This retention resulted in a reduction of the number of GCs that completely migrated out of the phalloidin-positive edge (Fig 1i). We also confirmed that Endo-N inhibited the migration of TuJ1^+^ and Prox1^+^ GCs (Fig 1j), which is consistent with Fig 1i.

**Fig 2:**
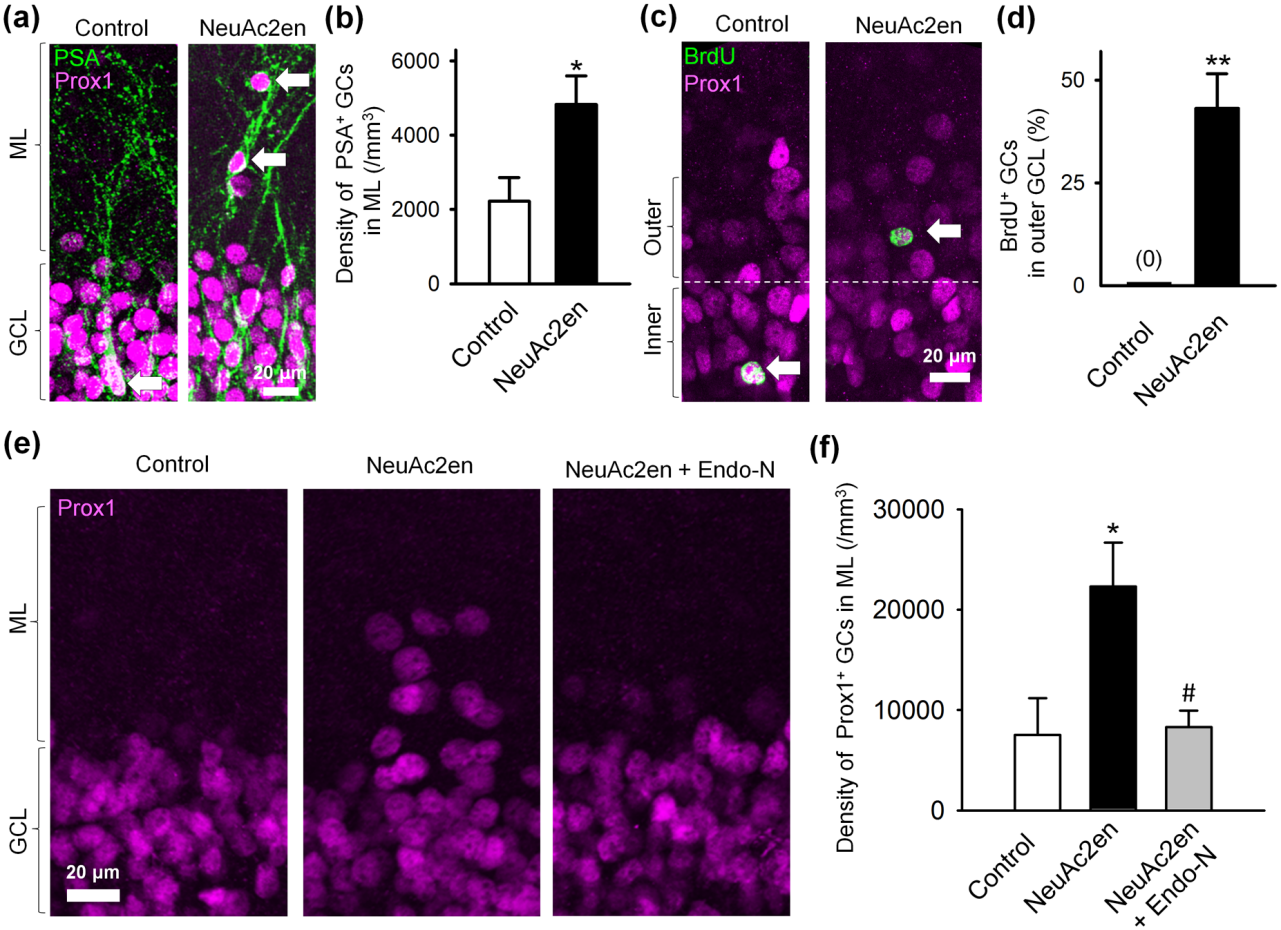
The involvement of neuraminidase in PSA degradation and GC positioning. (**a**) Representative confocal images of the GCL in cultured slices from P14 rats are shown. Samples were immunostained for PSA and Prox1 at 5 DIV. PSA^+^ immature GCs (arrows) were found ectopically in the molecular layer (ML) in NeuAc2en-treated cultures. (**b**) NeuAc2en significantly increased the density of PSA^+^ GCs in the ML. *P < 0.05, n = 4–6 slices. (**c**) Representative confocal images of the GCL in the cultured slices from P14 rats that received a BrdU injection at P13 are shown. Samples were immunostained for BrdU and Prox1 at 5 DIV. A dotted line, drawn down the middle of the GCL, indicates the boundary between the inner and outer GCL. (**d**) NeuAc2en significantly increases the percentage of BrdU^+^ GCs that exist in the outer GCL. The number of BrdU^+^ GCs in outer GCL is divided by the total number of BrdU^+^ GCs (defined as 100%) in the inner and outer GCL. **P < 0.01, n = 18–29 cells from 3–4 slices. (**e**, **f**) Representative confocal images of the GCL in slices cultured with NeuAc2en or NeuAc2en + Endo-N (**e**) and the density of Prox1+ GCs in the ML (**f**). The reagents were applied from 0 DIV and the cultures were fixed at 14 DIV and immunostained for Prox1. *P < 0.05 vs. control and ^#^*P* < 0.05 vs. NeuAc2en, n = 4 slices for each group.

The effects of Endo-N suggested that PSA facilitates GC migration. To examine the effects of PSA on GC lamination in the GCL, we utilized hippocampal slice cultures from P14 rats, and aimed to inhibit PSA degradation in the newly generated GCs. The mechanism of how PSA is cleaved from a neuron is unclear. We examined the role of the intrinsic neuraminidase/sialidases, which initiate the catabolism of sialo-glycoconjugates by removing their terminal sialic acid residues [21,22], in PSA degradation. We cultured the slices in the presence of NeuAc2en, a neuraminidase inhibitor. The location of immature GCs was examined by immunohistochemistry using anti-Prox1 and anti-PSA antibodies. In NeuAc2en-treated slices, the cell bodies of PSA^+^ GCs, in contrast to their normal location in the inner GCL in control cultures, were often found in the outer GCL and the molecular layer (Fig 2a). The density of the ectopic GCs in the molecular layer was significantly higher when compared with the control (Fig 2b). The ectopic GCs in NeuAc2en-treated slices (Fig 2a, arrows) possessed PSA^+^ leading process-like structures, suggesting that NeuAc2en inhibits PSA degradation and allows the immature GCs to aberrantly migrate into the molecular layer. To further examine the effects of NeuAc2en on newly generated GCs in their younger stages, we injected BrdU, an S-phase marker, into P13 rats to label proliferating GCs 24 h before preparing slice cultures. BrdU and Prox 1 double-positive GCs were located in the outer GCL in NeuAc2en-treated cultures and in the inner GCL under control conditions (Fig 2c and 2d). These results suggest that neuraminidase-dependent PSA degradation is required for the proper termination of GC migration and its lamination in the inner GCL. Finally, to prove that aberrant migration was caused by NeuAc2en through inhibiting PSA degradation, we needed to eliminate the possibility that cell migration was caused by non-specific effects of NeuAc2en. Thus, we tested if the effects of NeuAc2en on GC migration are blocked by removal of PSA with Endo-N. For this purpose, the hippocampal slices were cultured with both NeuAc2en and Endo-N (Fig 2e) and then the density of Prox1^+^ GCs in the molecular layer was examined. When both NeuAc2en and Endo-N were applied, the density of GCs in the molecular layer was not significantly different from control, which is in contrast to the results when NeuAc2en alone was applied (Fig 2e and 2f). We found that NeuAc2en application increased the density of GCs in the molecular layer (Fig 2e and 2f). Further, it seems that the density of cells in the granule cell layer were reduced compared to control (Fig 2e). We assume that these results indicate over-migration of young GCs from the GCL to the molecular layer. These idea are supported by the results that more BrdU-positive newborn GCs were found in the outer GCL in NeuAc2en-treated slice cultures compared to control (Fig 2c and 2d). Together, these results suggested that NeuAc2en impaired GC localization likely through inhibiting PSA degradation.

**Fig 3:**
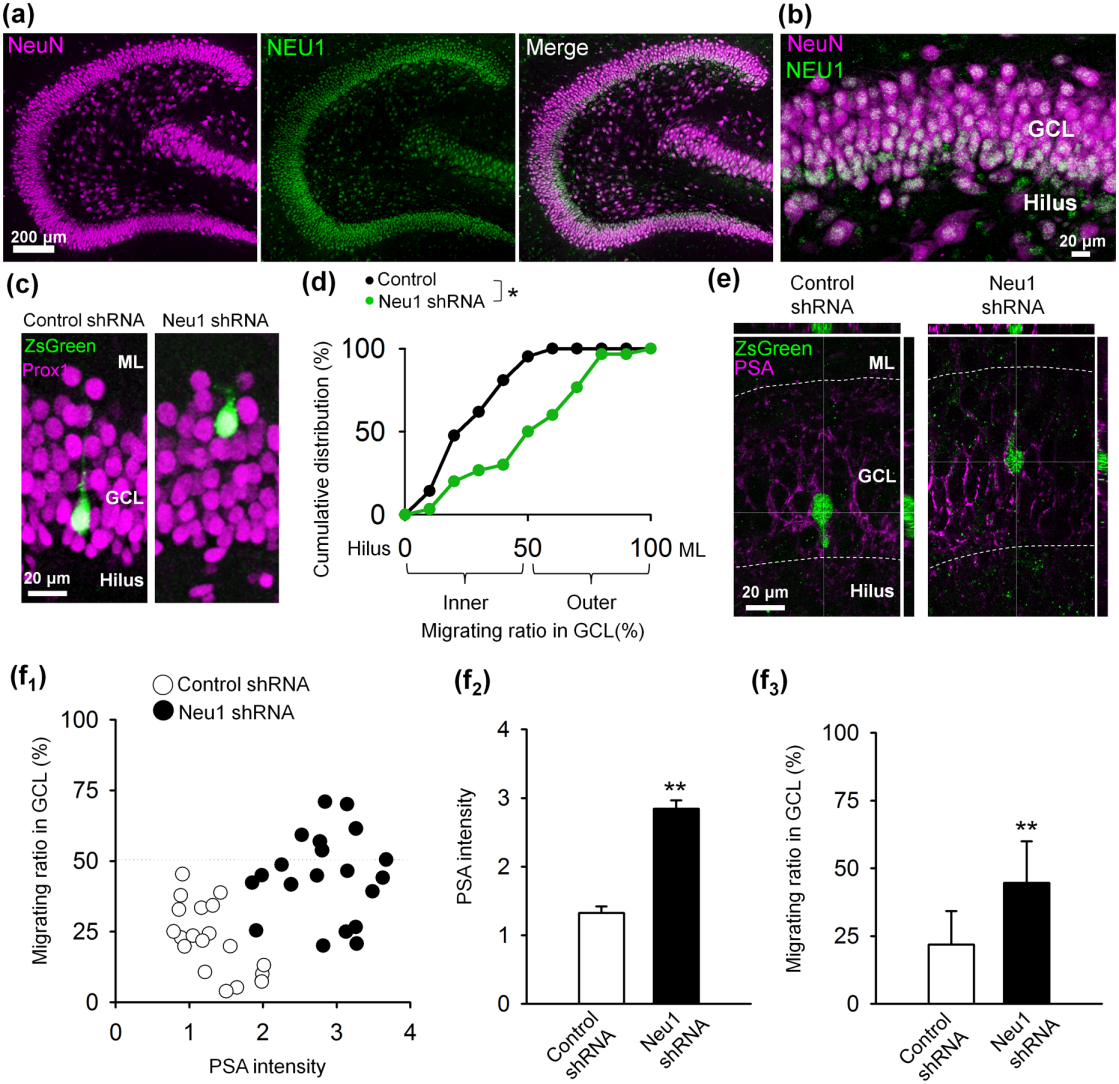
NEU1 regulates PSA presence and the positioning of newly generated GCs *in vivo*. Representative confocal images of a whole dentate gyrus (**a**) and the GCL (**b**) from a P14 rat hippocampal slice immunostained for NEU1 and the neuronal marker NeuN are shown. Immunohistochemical localization of NEU1 is higher in the innermost region of the GCL when compared with the middle and outer GCL (**b**). (**c**) Representative images of ZsGreen^+^ GCs in the GCL of P14 rats that received injections of retrovirus carrying either Control shRNA or Neu1 shRNA together with ZsGreen at P0 are shown. ML: molecular layer. (**d**) Cumulative distribution of the migrating ratio of shRNA-transfected GCs in the GCL (boundary to the hilus, 0%; boundary to the ML, 100%). The distribution of Neu1 shRNA-transfected GCs was significantly shifted toward the ML when compared with the Control shRNA-transfected GCs. *P < 0.05, Kolmogorov-Smirnov test, n = 20–29 cells. (**e**) Representative images of shRNA-transfected GCs immunostained for PSA at P14 are shown. (**f**) Neu1 shRNA-transfected GCs had higher levels of PSA (**f**_**1**_, **f**_**2**_) and were localized in the outer GCL (**f**_**1**_, **f**_**3**_). **P < 0.01, n = 20 cells.

The above results strongly suggest the involvement of intrinsic neuraminidase in the PSA-mediated regulation of GC lamination. Neuraminidases comprise a superfamily of intrinsic exoglycosidases that have a conserved active site and similar sequence motifs. Among them, NEU1 initiates the catabolism of sialo-glycoconjugates by removing their terminal sialic acid residues [23,24], and mutations in Neu1 gene cause sialidosis in humans [25]. Therefore, we examined the immunohistochemical localization of NEU1 in the P14 rat dentate gyrus (Fig 3), finding that NEU1 is localized in principal neurons, including GCs (Fig 3a). Importantly, we also found that NEU1 localization is higher in the innermost GCL, which contains immature GCs (Fig 3b). This high level of immunohistochemical localization of NEU1 in GCs implies the involvement of NEU1 in the development of newly generated GCs and the GCL.

**Fig 4:**
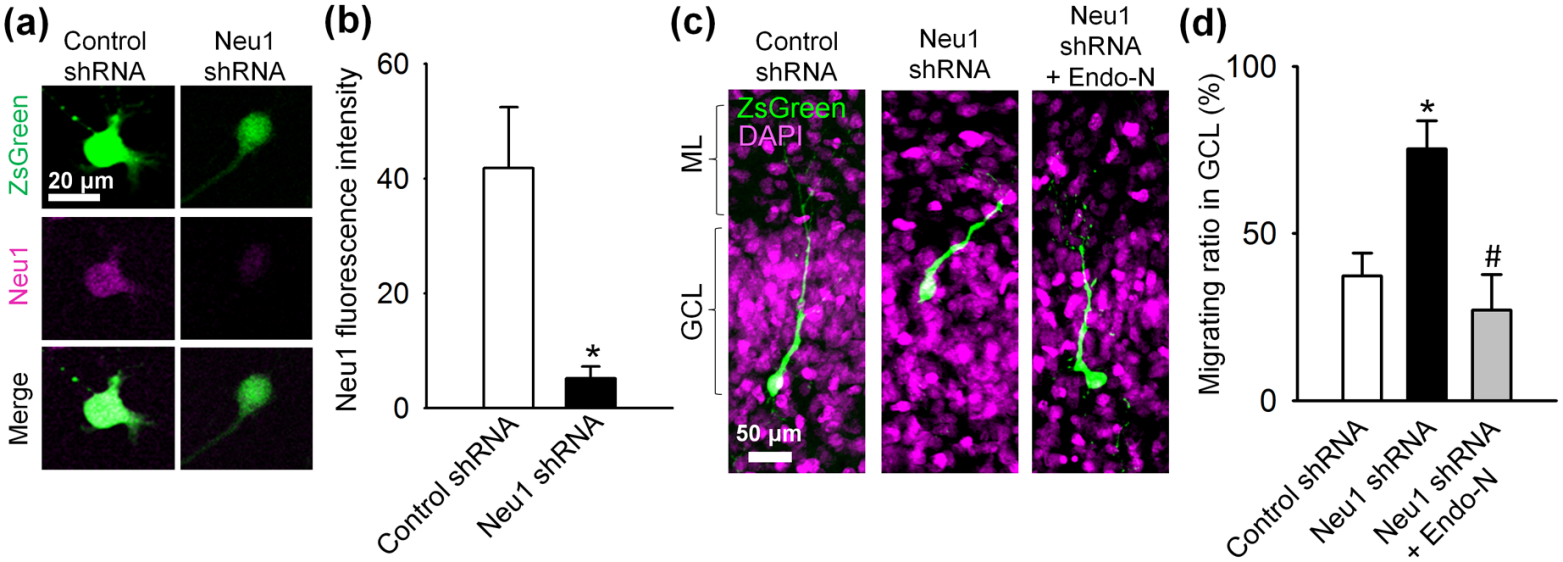
Verification of the effects of Neu1 shRNA in vitro. (**a**, **b**) The Neu1 shRNA-mediated knockdown of NEU1 was confirmed using immunocytochemistry in the primary culture of GCs prepared from P6 rats. GCs were transfected at 2 DIV with constructs encoding both shRNA and ZsGreen. Next, NEU1 immunofluorescence levels were examined at 5 DIV. Neu1 shRNA significantly decreases NEU1 intensity when compared with the scrambled control shRNA. (**c**) Representative confocal images of ZsGreen^+^ GCs in the GCL in cultured slices at 14 DIV. The retrovirus carrying either Control shRNA or Neu1 shRNA together with ZsGreen was dropped on the slice cultures at 0 DIV. In Neu1 shRNA + Endo-N cultures, Endo-N was applied from 0 DIV. (**d**) Bar graphs showing the migrating ratio of shRNA-transfected GCs in the GCL (boundary to the hilus, 0%; boundary to the ML, 100%).

The strong localization of NEU1 in GCs, especially in the innermost GCL, led us to test the idea that a newly generated GC regulates PSA localization with its own NEU1. For this purpose, we prepared constructs encoding short hairpin RNA sequences targeting rat Neu1 (Neu1 shRNA) and its scrambled control (Control shRNA) together with ZsGreen, a green fluorescent protein, as a reporter. The knockdown of NEU1 by these constructs was confirmed in the primary GC cultures (Fig 4a and 4b). Importantly, the migration ratio of GCs in the GCL was increased when NEU1 was knocked down with Neu1 shRNA in GCs in cultured hippocampal slices (Fig 4c and 4d): GCs retrovirally transfected with control shRNA located in the inner GCL, whereas GCs transfected with Neu1 shRNA frequently located in the outer GCL. Further, we examined off-target effects of Neu1 shRNA by removal of PSA with Endo-N. We found that GCs transfected with Neu1 shRNA localized in the proper region of GCL, i.e., the inner GCL, when the slices were cultured with Endo-N (Fig 4c and 4d; Neu1 shRNA + Endo-N). These results suggested that proper localization of GCs were regulated by their own NEU1. Next, we took advantage of retrovirus-mediated RNAi *in vivo* to infect proliferating newly generated GCs. Fourteen days after injecting a retrovirus encoding ZsGreen with Control or Neu1 shRNA into P0 rat pups, we found that most of the newly generated GCs that were transfected with Control shRNA were located in the inner GCL, whereas Neu1 shRNA-transfected GCs tended to be present in the outer GCL (Fig 3c–3f_3_). Importantly, GCs that had NEU1 knocked down exhibited a higher level of PSA presencewhen compared with control GCs (Fig 3e, 3f_1_ and 3f_2_). These results suggest that NEU1 cleaves PSA from newly generated GCs in a cell autonomous fashion and terminates their migration.

## Discussion

The strong localization of PSA in immature GCs is well recognized, but the role of PSA in GC development and the mechanism of how PSA presence is regulated is unclear. In the adult dentate gyrus, where the clustering of PSA^+^ immature GCs are observed [26], removing PSA by injecting Endo-N *in vivo* increased the number of newly generated cells inside the cluster. These results imply that PSA facilitates the migration of newly generated cells away from the clusters [27]. In the present study, a novel explant culture system of the hilus directly revealed that the enzymatic removal of PSA attenuates the migration of immature GCs (Fig 1c–1j). Furthermore, we have shown that NEU1 in newly generated GCs cell-autonomously regulates PSA presence. In the present study, we mainly performed immunohistochemical and immunocytochemical analysis to determine the presence and role of PSA on migrating GCs. Thus, the present study have not focused on determining whether and how the expression levels of PSA affect GC migration. One of the most important conclusions in the present study is that the presence of PSA, but not the expression levels of PSA, on the young GC surface facilitates their migration. Indeed, it has been suggested that the presence of PSA on the cell surface, but not inside a cell, is crucial for cell-cell interaction and cell migration, especially because PSA is a large volume sugar chain [10].

It was previously unclear how PSA presence is regulated. Considering that PSA is involved in multiple and continuous forms of structural plasticity, such as cell migration and positioning, neurite growth, and synaptic plasticity [28], tightly regulated spatiotemporal levels of PSA localization are critical for establishing the proper neural circuitry. Two major possible regulators of cell-surface PSA levels have been suggested [28]: the synthesis of PSA-NCAM through the activity of sialyltransrerases and the turnover of the molecule at the cell surface. Our study provides a novel mechanism that consists of the cell-intrinsic, NEU1-meditated decrease in PSA presence during neuronal migration, which is required for the termination of migration and the proper positioning of neurons. NEU1 has been extensively studied as a target gene for sialidosis, but its role in the central nervous system has not been fully elucidated [29]. Future studies are necessary to examine the involvement of other neuraminidases in the structural plasticity of the brain.

Our data suggest that the lamination of GCs is tightly regulated by PSA presence. However, it is not clear what purpose GC lamination serves in this brain region. We find one possible answer in the highly laminated structure of the dentate gyrus [5]. In the molecular layer where GC dendrites receive synaptic inputs, layer-specific projections from different neuronal cell types result in developmental stage-dependent synaptic inputs that would contribute to the proper differentiation of newly generated GCs [30]. Proper GC lamination is also important from a pathological viewpoint because GCs were found to be dispersed in epileptic brains [6].

In summary, our data link the regulation of PSA levels on the surface of newly generated GCs to the proper positioning of these cells in the brain. Importantly, we discovered a novel role of cell-intrinsic NEU1 in the regulation of PSA presence during GC lamination.
